# The Effects of 1-*O*-Acetylbritannilactone Isolated from *Inula britannica* Flowers on Human Neutrophil Elastase and Inflammation of RAW 264.7 Cells and Zebrafish Larvae

**DOI:** 10.3390/molecules28114320

**Published:** 2023-05-24

**Authors:** Ik Soo Lee, Yu-Ri Lee, Jea Heon Sim, Ki Mo Kim, Young Sook Kim

**Affiliations:** 1 Korean Medicine Convergence Research Division, Korea Institute of Oriental Medicine, Daejeon 34054, Republic of Korea; knifer48@kiom.re.kr (I.S.L.); yurilee@kiom.re.kr (Y.-R.L.); 2 Department of Oriental Medicinal Biotechnology, College of Life Sciences, Kyung Hee University, Yongin 17104, Republic of Korea; tla7370@naver.com

**Keywords:** *Inula britannica*, sesquiterpene lactone, human neutrophil elastase, RAW 264.7 cells, zebrafish

## Abstract

During a search for natural inflammatory inhibitors, 1-*O*-acetylbritannilactone (ABL), a sesquiterpene lactone, was isolated from the flowers of *Inula britannica*. ABL significantly inhibited human neutrophil elastase (HNE) with a half-maximal inhibitory concentration (IC_50_) of 3.2 ± 0.3 µM, thus did so more effectively than the positive control material (epigallocatechin gallate) (IC_50_ 7.2 ± 0.5 µM). An enzyme kinetic study was performed. ABL noncompetitively inhibited HNE with an inhibition constant *K*_i_ of 2.4 µM. ABL inhibited lipopolysaccharide-induced nitric oxide and prostaglandin E_2_ production by RAW 264.7 cells in a dose-dependent manner, as well as the protein-level expression of inducible nitric oxide synthase and cyclooxygenase-2. The anti-inflammatory effect of ABL was confirmed using a transgenic Tg(*mpx*:EGFP) zebrafish larval model. The exposure of the larvae to ABL inhibited neutrophil recruitment to the site of injury after tail fin amputation.

## 1. Introduction

Polymorphonuclear neutrophils (PMNs) are the most abundant circulating leukocytes in human blood and they play crucial roles in the innate immune system [1,2]. Normally, the most common PMNs are neutrophils, produced in large quantities in the venous sinuses of bone marrow to serve as the first line of immune defense against invading pathogens via phagocytosis, degranulation, and the release of neutrophil extracellular traps [1]. Low neutrophil numbers or dysfunctional neutrophils trigger various disorders, of which neutropenia is the most common and which results from a decreased neutrophil production or increased destruction [3]. These functional disorders include impaired neutrophil chemotaxis, adhesion, phagocytosis, and bactericidal activity [4].

Human neutrophil elastase (HNE; EC 3.4.21.37) is a 29-kDa serine protease of the chymotrypsin family located in the azurophilic granules of PMNs [5] that is released during neutrophil degranulation [6]. HNE plays a critical role in immune system physiology [7]. Intracellular HNE breaks down invading bacteria; HNE secreted by neutrophils aids cell migration to sites of inflammation by degrading extracellular macromolecules, including collagen, elastin, and keratin [8,9,10]. Under normal physiological conditions, HNE activity is controlled by endogenous protease inhibitors such as the α1-protease and secretory leukocyte protease inhibitors [8,9]. However, large amounts of proteases released by leukocytes recruited to the sites of inflammation may inactivate such endogenous inhibitors [11]. Such an imbalance between HNE and endogenous inhibitors can lead to the development of chronic inflammatory diseases including rheumatoid arthritis, adult respiratory destress syndrome, and cystic fibrosis [8,11,12]. Natural pharmacological HNE inhibitors might prevent and treat inflammation-related diseases [8,13].

We previously found that an *Inula britannica* flower extract exhibited considerable HNE inhibitory activity [14]. A phytochemical study revealed that the sesquiterpene lactone 1-O-acetylbritannilactone (ABL) was the major active compound. Here, we investigate the HNE inhibitory activity of ABL, and the effects of ABL on inflammation of lipopolysaccharide (LPS)-induced RAW 264.7 macrophages in vitro, and transgenic Tg(*mpx*:EGFP) zebrafish larvae in vivo.

## 2. Results

### 2.1. Isolation and Identification of ABL

**Figure 1:**
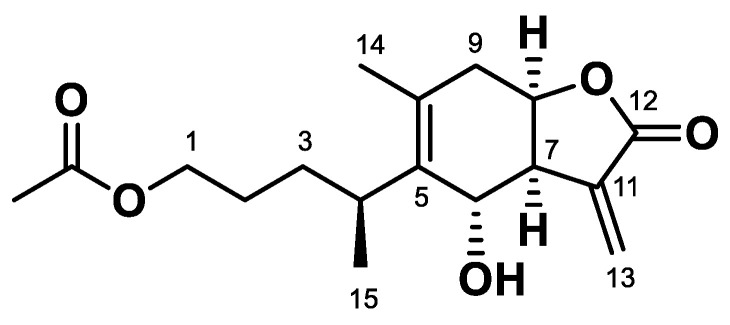
Chemical structure of ABL from the flowers of *I. britannica*.

An ethanol extract of *I. britannica* flowers was subjected to a series of chromatographic separations guided by HNE inhibitory activity. This yielded an amorphous powder C_17_H_24_O_5_, as established by electrospray ionization-mass spectrometry (ESI-MS), based on a molecular ion peak at *m*/*z* 309 [M + H]^+^ (Figure S1). The infrared spectrum exhibited the characteristic absorption bands of hydroxy (3495 cm^−1^), carbonyl (1728 and 1722 cm^−1^), and olefinic (1640 cm^−1^) moieties (Figure S3). The ^1^H-NMR spectrum evidenced the signals characteristic of exocyclic methylenes at H-13a and H-13b (δ_H_ 6.23 and 5.83 for both d values, *J* = 2.4 Hz); an acetoxyl group at δ_H_ 2.01 (3H, s); and a branched pentanol moiety (Figure S4). The ^13^C-NMR spectrum revealed 17 carbons, of which the signals at δ_C_ 172.5 (C=O, C-12), 139.1 and 124.8 (C=CH_2_, C-11 and C-13), and 78.5 (C-8) were assigned to an α-methylene-γ-lactone moiety. The signals at δ_C_ 173.2 and 21.0 were assigned to an acetoxy group (Figure S4). Finally, the compound was identified as ABL by comparing the spectral data to those in the literature [15] (Figure 1).

### 2.2. HNE Inhibitory Activity of ABL

The in vitro inhibition of HNE by ABL was evaluated as described previously [16]. ABL inhibited HNE catalysis in a dose-dependent manner with a half-maximal inhibitory concentration (IC_50_) of 3.2 ± 0.3 µM, and was indeed more inhibitory than the positive control (epigallocatechin gallate) (IC_50_ 7.2 ± 0.5 µM). To further characterize HNE inhibition, the concentrations of ABL and the substrate were varied. The oxidation of HNE by ABL followed Michaelis–Menten kinetics. The Lineweaver–Burk plot showed that the 1/*V*_max_ values increased gradually with increasing concentrations of ABL (from 0 to 5 μM), whereas the x-intercept (−1/*K*_m_) was unaffected (Figure S5A), indicating that ABL inhibited HNE in a noncompetitive manner. The Dixon plot revealed that the inhibition constant *K*_i_ of ABL was 2.4 µM (Figure S5B).

### 2.3. Effect of ABL on RAW 264.7 Cell Viability

**Figure 2:**
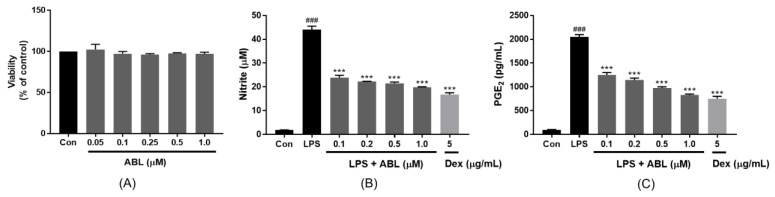
The effects of ABL on LPS-induced NO and PGE_2_ production by RAW 264.7 cells. Cells were incubated for 24 h with various concentrations of ABL (0.05–1.0 μM) and (**A**) cell viability was assessed using the MTS assay. Cells were stimulated with 1 µg/mL LPS for 24 h after pretreatment with the indicated concentrations of ABL (0.1, 0.2, 0.5, and 1.0 μM) and Dex (5 μg/mL). (**B**) NO production was determined using the Griess reagent system. (**C**) PGE_2_ concentrations in supernatants were measured with the aid of an ELISA kit. The values are the means ± SDs of those from three independent experiments. ^###^ *p* < 0.001 vs. Control, *** *p* < 0.001 vs. LPS.

To explore whether ABL affected cell viability, RAW 264.7 cells were incubated for 24 h with various concentrations of ABL (0.05–1.0 μM), and cell viability was assessed using the MTS assay. ABL did not affect viability (Figure 2A). Under the microscope, no cell morphological changes were observed. Thus, known non-toxic concentrations (up to 1.0 μM) were used in subsequent experiments.

### 2.4. Effect of ABL on LPS-Induced NO and PGE_2_ Production

The overproduction of pro-inflammatory mediators, such as nitric oxide (NO) and prostaglandin E2 (PGE_2_), is a hallmark of macrophage-mediated inflammation [17]. To explore whether ABL inhibited NO and PGE_2_ production by LPS-induced RAW 264.7 cells, the cells were treated with various concentrations of ABL (0.1, 0.2, 0.5, and 1.0 μM) and then incubated with LPS (1 μg/mL) for 24 h. As shown in Figure 2B,C, NO and PGE_2_ production increased significantly in the LPS-treated control group but ABL pretreatment inhibited these increases in a dose-dependent manner with IC_50_ values of 0.23 ± 0.02 and 0.27 ± 0.02 µM, respectively. This activity can be compared with other naturally occurring sesquiterpene lactones. Ergolide, isolated from *I. britannica*, inhibited the LPS/IFN-γ-mediated production of NO and PGE_2_ with IC_50_ values of 1.95 and 3.0 µM, respectively [18]. Seven sesquiterpene lactones (costunolide, dehydrocostus lactone, eremanthine, zaluzanin C, magnolialide, santamarine, and spirafolidc), isolated from the leaves of *Taurus nobifis*, reduced LPS-induced NO production with IC_50_ values of 1.2–3.8 µM [19].

### 2.5. Effect of ABL on LPS-Induced iNOS and COX-2 Protein Expression Levels

**Figure 3:**
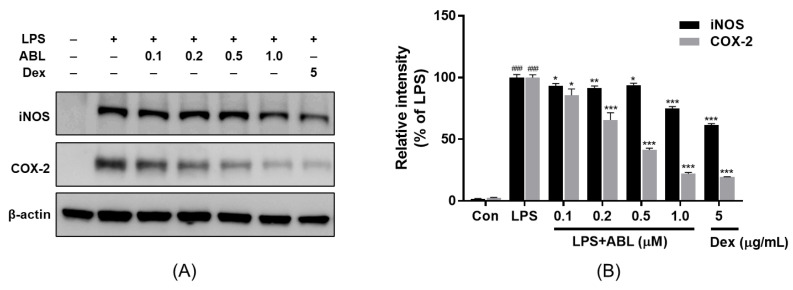
The effects of ABL on iNOS and COX-2 protein expression levels in LPS-induced RAW 264.7 cells. Cells were stimulated with 1 µg/mL LPS for 24 h after pretreatment with the indicated concentrations of ABL (0.1, 0.2, 0.5, and 1.0 μM) and Dex (5 μg/mL). Equal concentrations of protein (20 µg) were separated via 4–20% (*w*/*v*) SDS-PAGE. (**A**) iNOS and COX-2 proteins were detected via Western blotting using specific anti-iNOS and anti-COX-2 antibodies, and β-actin served as a loading control. (**B**) The protein band intensities were quantified using ImageJ 1.53k software. The values are the means ± SDs of those from three independent experiments. ^###^ *p* < 0.001 vs. Control, * *p* < 0.05 vs. LPS, ** *p* < 0.01 vs. LPS, *** *p* < 0.001 vs. LPS.

As the pro-inflammatory mediators NO and PGE_2_ are synthesized by inducible nitric oxide synthase (iNOS) and cyclooxygenase-2 (COX-2), respectively [20], we measured the levels of these enzymes in LPS-induced RAW 264.7 cells via Western blotting. As shown in Figure 3A, LPS greatly increased the expression levels of both proteins, and ABL pretreatment suppressed these rises in a dose-dependent manner. ABL at 0.5 and 1.0 μM reduced COX-2 protein expression by about 59.1% and 77.9%, respectively, compared to the LPS-treated control group, but ABL inhibited iNOS protein expression to a much lesser extent (Figure 3B).

### 2.6. Effect of ABL on Neutrophil Migration in Injured Zebrafish Larvae

**Figure 4:**
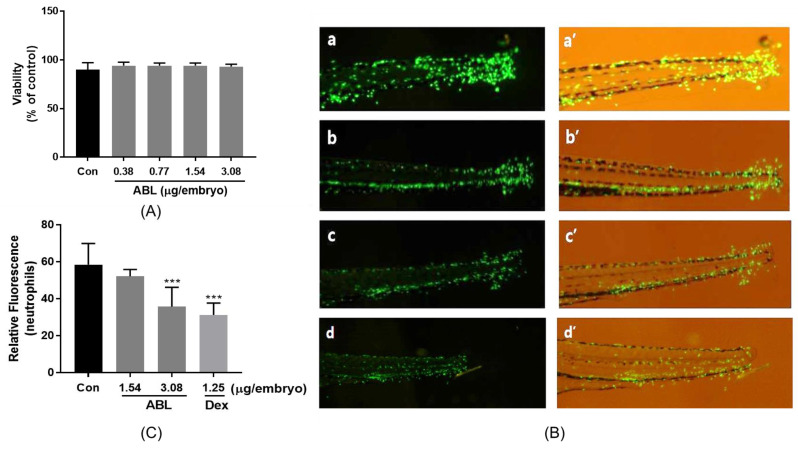
The effect of ABL on neutrophil migration after injury of Tg(*mpx*:EGFP) zebrafish larvae. (**A**) Viability of zebrafish embryos after exposure to various doses of ABL (0.38–3.08 μg/embryo) for 24 h. After tail fin amputation, zebrafish larvae were exposed to 1.54 and 3.08 μg/embryo of ABL or Dex (1.25 μg/embryo), and the neutrophil fluorescence intensities at the injury site were measured at 24 hpi. (**B**) A representative fluorescence image of zebrafish larvae treated with ABL (a and a′ 0 μg/embryo; b and b′, 1.54 μg/embryo; c and c′ 3.08 μg/embryo) and Dex (d and d′ 1.25 μg/embryo). (**C**) The fluorescence intensities were quantified using ImageJ 1.53k software. The values are the means ± SEs of those from three independent experiments. *** *p* < 0.001 vs. Control.

We investigated the anti-inflammatory effect of ABL after the tail fin amputation of zebrafish larvae. ABL was not toxic to zebrafish embryos at doses up to 3.08 μg/embryo (Figure 4A). Thus, when evaluating the effect of ABL on neutrophil migration, we treated larvae at 5 days post-fertilization (dpf) with 1.54 and 3.08 μg/embryo of ABL immediately after fin amputation and measured the neutrophil fluorescence intensities at the site of injury at 24 h post-injury (hpi). As shown in Figure 4B, the number of neutrophils that migrated to the injury site greatly increased in the untreated control, but ABL treatment inhibited migration in a dose-dependent manner. The neutrophil intensity at the injury site decreased by approximately 38.9% on treatment with 3.08 μg/embryo of ABL, compared to the untreated control group; the ABL effect was comparable to that of dexamethasone (Dex; 40.0% inhibition at 1.25 μg/embryo) (Figure 4C).

## 3. Discussion

Natural products have been used for thousands of years to treat human ailments; such products greatly aid innovative drug discovery. Some medicinal plants serve as anti-inflammatory agents [21]. Thus, the secondary metabolites of such plants may serve as valuable leads for drug development targeting inflammation-related diseases.

*I. britannica* (Asteraceae) is one of the most commonly used traditional Chinese and Kampo medicines for various diseases, including inflammation [22]. Many secondary metabolites, including steroids, terpenoids (sesquiterpenes, diterpenes, and triterpenoids), phenolics, and flavonoids are known from the plant [23]. Of these, sesquiterpenes, especially sesquiterpene lactones, are both the most characteristic and most abundant components of *Inula*; the biological properties include cytotoxic, apoptotic, and anti-inflammatory activities [23]. Here, we found that ABL, a sesquiterpene lactone with an α-methylene-γ-lactone moiety, was the major HNE inhibitory compound in an ethanol extract of *I*. *britannica* flowers. HNE stimulates various pro-inflammatory signaling pathways via multiple mechanisms [24]. In vitro, ABL dose-dependently inhibited LPS-induced NO and PGE_2_ production in RAW 264.7 cells. Consistent with this finding, ABL also suppressed iNOS and COX-2 protein expression.

The zebrafish is widely used to screen for novel anti-inflammatory agents. Most studies evaluate the neutrophil and macrophage responses of zebrafish using transgenic lines with fluorescent neutrophils, fluorescent macrophages, or both [25,26]. The Tg(*mpx*:EGFP) zebrafish is a transgenic zebrafish that expresses enhanced green fluorescent protein (EGFP) in only neutrophils; the model is widely used to study inflammation and immune responses [25]. To confirm the anti-inflammatory effect of ABL in vivo, we used transgenic zebrafish expressing EGFP-tagged neutrophils. ABL inhibited neutrophil recruitment to the injury induced by tail fin amputation, in line with our in vitro enzyme (HNE) and cell-based (RAW 264.7 cells) data, suggesting that ABL exerts an anti-inflammatory effect.

This study suggests that HNE is an important regulator of inflammation and that ABL, or derivatives thereof, may usefully treat inflammation-related diseases by inhibiting HNE and other pro-inflammatory mediators.

## 4. Materials and Methods

### 4.1. General Experimental Procedures

Ultraviolet (UV) and infrared (IR) spectra were recorded using JASCO V-550 UV/visible and JASCO 100 IR spectrometers (JASCO, Tokyo, Japan), respectively. ^1^H (400 MHz) and ^13^C NMR (100 MHz) spectra were obtained using a Bruker DRX-400 spectrometer (Bruker, Billerica, MA, USA) with tetramethylsilane as the internal standard. ESI-MS was performed using a Shimadzu liquid chromatograph ion-trap (IT) and time-of-flight (TOF) LCMS-IT-TOF mass spectrometer (Shimadzu, Kyoto, Japan). Column chromatography employed Diaion HP-20 (Supelco, St. Louis, MO, USA) and YMC-gel ODS-A (S-75 µm; YMC, Kyoto, Japan) systems. Thin-layer chromatography was performed on pre-coated silica gel 60 F_254_ (0.25 mm; Merck, Rahway, NJ, USA) and RP-18 F_254s_ plates (0.25 mm; Merck). Spots were detected under UV light (254 nm), via spraying of 10% (*v*/*v*) H_2_SO_4_, followed by heating.

### 4.2. Plant Material

Flowers of *I. britannica* were purchased from a traditional herbal medicine store in Daejeon, Republic of Korea, in August 2016, and identified by Prof. Ki Hwan Bae (College of Pharmacy, Chungnam National University, Republic of Korea). A voucher specimen (IB2016-001) has been deposited in the Herbarium of the Korean Institute of Oriental Medicine, Republic of Korea.

### 4.3. Extraction and Isolation

Dried flowers of *I. britannica* (1 kg) were extracted into ethanol (10 L) at 80 °C for 3 h, the extract filtered, and then concentrated to yield an ethanol extract (110 g) that was subjected to Diaion HP-20 column chromatography (60 × 10 cm) using a gradient solvent system of methanol and water (100% water → 100% methanol). The resulting fractions were combined into five (A–E), based on the TLC results. In this bioassay-guided study, the most active column fraction C was chromatographed on a YMC-gel ODS-A column (60 × 6.5 cm) using a methanol–H_2_O gradient solvent system (20:80 → 100:0) to yield four subfractions (C1–C4). Fraction C2 was further chromatographed on a YMC-gel ODS-A column (50 × 4 cm) using a methanol–H_2_O gradient (20:80 → 80:20) to generate five subfractions (C2.1–C2.5). Finally, fraction C2.3 was chromatographed on a YMC-gel ODS-A column (60 × 2.5 cm) using a methanol–H_2_O gradient (60:40 → 80:20) to yield ABL (38 mg, 0.035% yield).

*1-O-Acetylbritannilactone (ABL).* white amorphous powder; UV (MeOH) λ_max_ 210 nm; IR (KBr) *ν*_max_ 3495, 2926, 1728, 1722, 1640, 1412, 1352, 1260, 1029 cm^−1^; ESI-MS *m*/*z* 309 [M + H]^+^; ^1^H-NMR (CD_3_OD, 400 MHz): δ 6.23 (1H, d, *J* = 2.4 Hz, H-13a), 5.83 (1H, d, *J* = 2.4 Hz, H-13b), 5.06 (1H, m, H-8), 4.15 (1H, d, *J* = 2.0 Hz, H-6), 3.90 (2H, m, H-1), 3.50 (1H, m, H-7), 2.86 (1H, dd, *J* = 16.0, 2.4 Hz, H-9a), 2.72 (1H, m, H-4), 2.40 (1H, dd, *J* = 16.0, 2.0 Hz, H-9b), 2.01 (3H, s, COCH_3_), 1.74 (3H, s, CH_3_-14), 1.07 (3H, d, *J* = 7.2 Hz, CH_3_-15); ^13^C-NMR (CD_3_OD, 100 MHz): δ 173.2 (COCH_3_), 172.5 (C-12), 139.1 (C-11), 137.6 (C-5), 131.5 (C-10), 124.8 (C-13), 78.5 (C-8), 68.9 (C-6), 65.7 (C-1), 47.4 (C-7), 35.5 (C-9), 34.4 (C-4), 32.7 (C-3), 28.0 (C-2), 21.0 (COCH_3_), 20.6 (C-14), 19.5 (C-15).

### 4.4. HNE Inhibitory Assay

HNE inhibitory activity was evaluated as described previously [16]. Briefly, reaction mixtures (100 µL) containing 10 mM Tris-HCl buffer (pH 7.5), 1.0 mM MeO-Suc-Ala-Ala-Pro-Val-*p*-nitroanilide (Sigma, St. Louis, MO, USA), 0.18 U HNE (CalbioChem, San Diego, CA, USA), and various concentrations of ABL were incubated in a 96-well plate for 1 h at 37 °C in the dark. Each reaction was stopped by the addition of 100 µL soybean trypsin inhibitor (0.2 mg/mL; CalbioChem, San Diego, CA, USA) and the absorbance at 405 nm immediately measured using a microplate reader. Epigallocatechin gallate (Sigma, St. Louis, MO, USA) served as the positive control. All experiments were performed in triplicate. IC_50_ values were estimated from the least-squares regression lines of the logarithmic concentrations plotted against the remaining activities, using GraphPad 7.0 Prism software (GraphPad Software, Inc., Chicago, IL, USA).

### 4.5. Kinetic Analysis

The kinetic study employed the above medium in which the ABL concentrations varied and the substrate concentrations ranged from 0.1 to 0.8 mM. Each reaction commenced on addition of diluted substrate, and data were recorded every 2 min for 10 min at 37 °C. The maximum velocity (*V*_max_) and Michaelis constant (*K*_m_) were determined by drawing Lineweaver–Burk plots. The inhibition constant (*K*_i_) was calculated from the Dixon plot. All graphs were plotted using SigmaPlot 12.5 software (Systat Software, Inc., Chicago, IL, USA).

### 4.6. Cell Culture

RAW 264.7 cells were from the American Type Culture Collection (ATCC, Manassas, VA, USA) and were maintained in Dulbecco’s modified Eagle’s medium (Gibco, Thermo Fisher Scientific, Waltham, MA, USA) supplemented with 10% (*v*/*v*) fetal bovine serum (Gibco, Thermo Fisher Scientific) and 1% (*w*/*v*) penicillin/streptomycin at 37 °C in a humidified 5% (*v*/*v*) CO_2_ incubator.

### 4.7. Cell Proliferation

RAW 264.7 cells were plated in 96-well plates at 1 × 10^4^ cells/well and incubated for 24 h. The medium was then replaced with fresh medium containing various concentrations of materials, followed by incubation for 24 h. The cell proliferation rate was measured using the 3-(4,5-dimethylthiazol-2-yl)-5-(3-carboxymethoxyphenyl)-2-(4-sulfophenyl)-2H-tetrazolium inner salt (MTS) assay (Promega, Madison, WI, USA), according to the manufacturer’s protocol.

### 4.8. Measurement of NO and PGE_2_ Production

RAW 264.7 cells were plated in 12-well plates at 5 × 10^4^ cells/well and incubated for 24 h. The cells were then stimulated with 1 µg/mL LPS for 24 h after pretreatment with various materials. The culture supernatants were harvested and NO production determined via assay of nitrite levels using the Griess reagent system (Promega, Madison, WI, USA) according to the manufacturer’s protocol. PGE_2_ concentrations in supernatants were measured via enzyme-linked immunosorbent assay (ELISA) (Cayman Chemical, Ann Arbor, MI, USA) according to the manufacturer’s protocol. Dex (Sigma, 5 μg/mL) served as the positive control.

### 4.9. Western Blotting

Cell lysates were prepared as described previously [27]. Protein concentrations were measured using a bicinchoninic acid protein assay kit (Pierce Biotechnology, Waltham, MA, USA). Equal amounts of protein (20 µg) were separated via 4–20% (*w*/*v*) sodium dodecyl sulfate–polyacrylamide electrophoresis (SDS-PAGE) and transferred to polyvinylidene difluoride membranes. After blocking, the membranes were incubated with antibodies against β-actin, iNOS, and COX-2 (Cell Signaling Technology, Danvers, MA, USA) and then incubated with horseradish peroxidase-conjugated secondary antibodies (Cell Signaling Technology, Danvers, MA, USA); bands were developed using an enhanced chemiluminescence detection system (Amersham Biosciences, Amersham, UK). Protein levels were determined with the aid of an LAS-4000 image analyzer (Fujifilm, Tokyo, Japan) running Image Gauge 3.0 software (Fujifilm, Tokyo, Japan).

### 4.10. Zebrafish Strains and Maintenance

Tg(*mpx*:EGFP) zebrafish were maintained in a facility of the Korean Institute of Oriental Medicine at 28.5 °C under a 14 h light/10 h dark cycle. Embryos were naturally produced by Tg(*mpx*:EGFP) zebrafish and grew freely in egg water.

### 4.11. Zebrafish Neutrophil Recruitment Assay

Tg(*mpx*:EGFP) zebrafish larvae at 5 dpf were randomly divided into five groups and placed into 6-well plates (*n* = 20–25 per group). The larvae were anesthetized with tricaine (Sigma) and the lower parts of the tails amputated using a scalpel. After injury, the larvae were exposed to ABL (1.54 and 3.08 μg/embryo) or Dex (1.25 μg/embryo) at 28.5 °C. The test materials were dissolved in dimethyl sulfoxide and directly pipetted into egg water with the larvae. Fluorescence images at 24 hpi were obtained using a stereomicroscope (SZX16; Olympus, Tokyo, Japan). The fluorescence intensities were quantified with the aid of ImageJ 1.53k software.

### 4.12. Statistical Analysis

Statistical significance was assessed using one-way analysis of variance and Dunnett’s multiple comparison test in GraphPad 7.0 Prism software (GraphPad Software, Inc., San Diego, CA, USA).

## Data Availability

Not applicable.

## Supplementary Materials

The following supporting information can be downloaded at: https://www.mdpi.com/article/10.3390/molecules28114320/s1. Figure S1: ESI-MS of ABL; Figure S2: UV spectrum of ABL; Figure S3: IR spectrum of ABL; Figure S4: NMR spectrum of ABL in CD_3_OD (400 MHz for ^1^H NMR, 100 MHz for ^13^C NMR); Figure S5: Kinetic study on HNE inhibition by ABL.
