# Supplementary material for: The Effects of 1-O-Acetylbritannilactone Isolated from Inula britannica Flowers on Human Neutrophil Elastase and Inflammation of RAW 264.7 Cells and Zebrafish Larvae
Source: Molecules. 2023 May 24;28(11):4320. doi: 10.3390/molecules28114320 (PMC10254531; doi:10.3390/molecules28114320)
Supplement: Supplementary file 1 [file molecules-28-04320-s001.zip › molecules-2403641-supplementary.pdf]

Supporting information

The Effects of 1-*O*-Acetylbritannilactone Isolated from *Inula britannica* Flowers on Human Neutrophil Elastase and In-flammation of RAW 264.7 Cells and Zebrafish Larvae

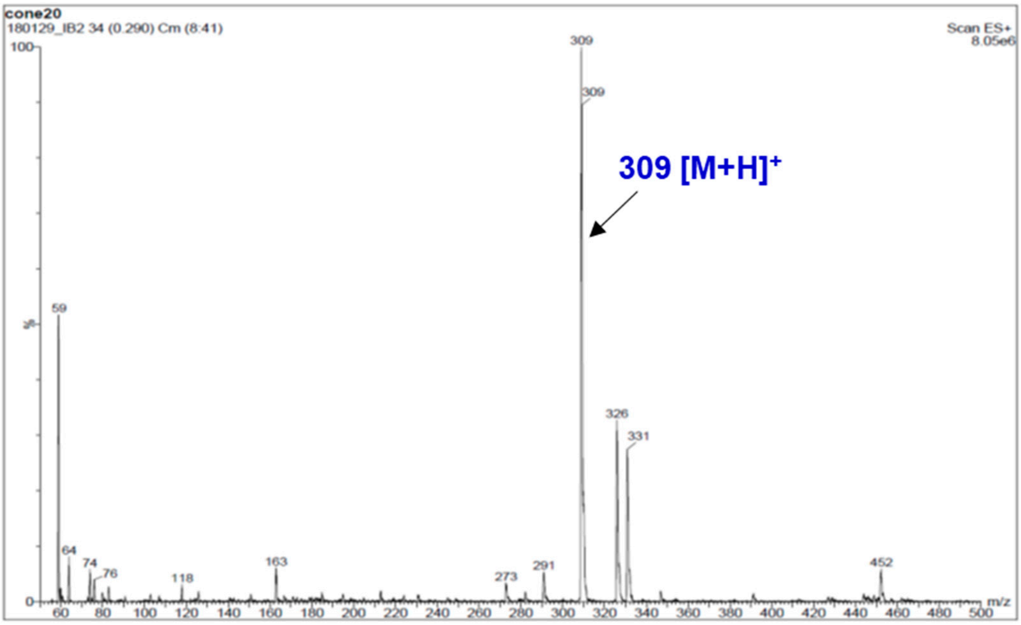

Figure S1. ESI-MS of ABL

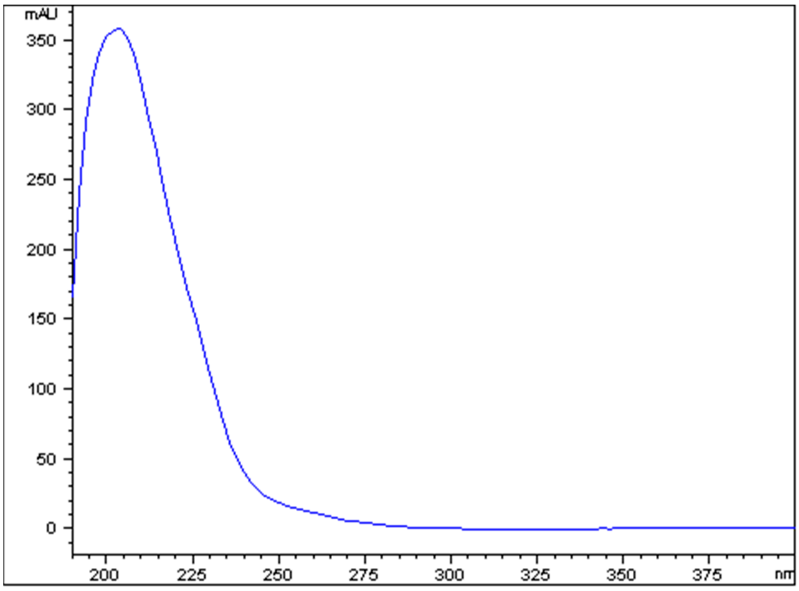

Figure S2. UV spectrum of ABL

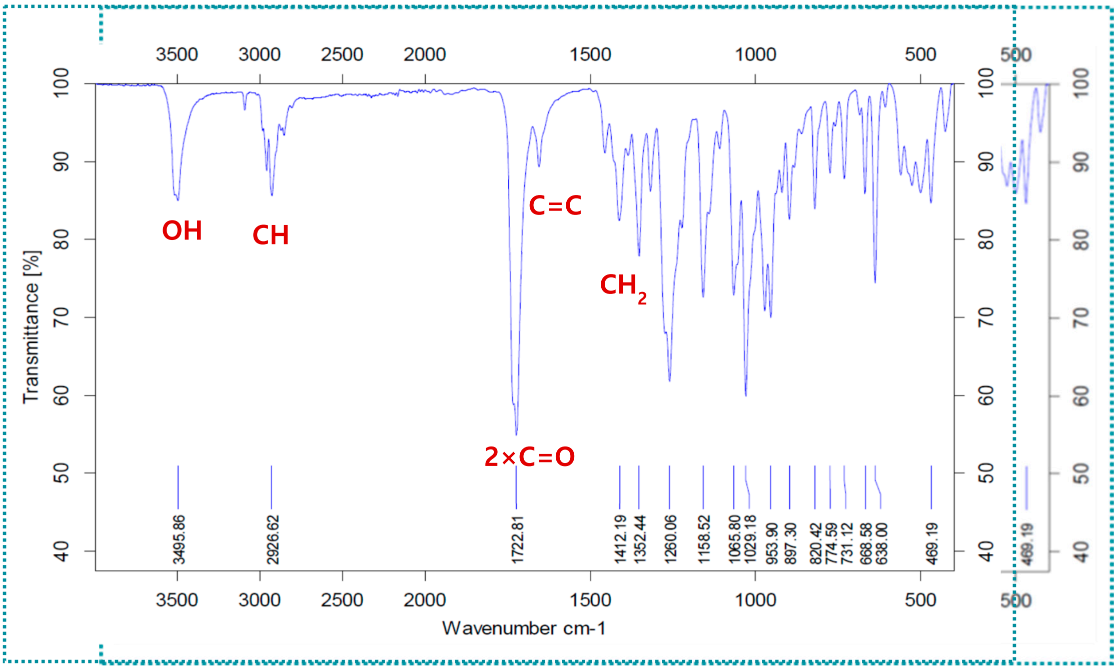

Figure S3. IR spectrum of ABL

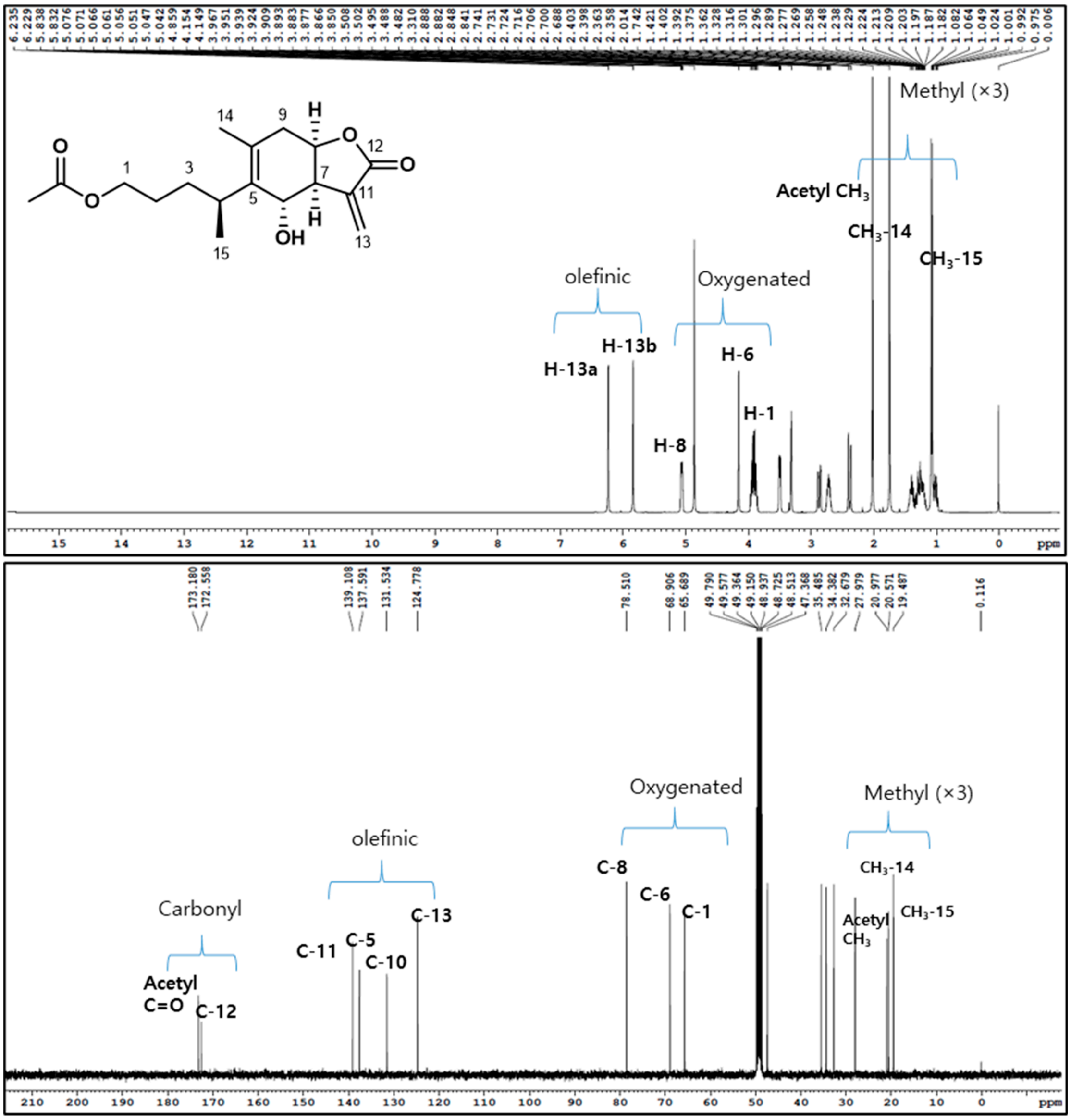

Figure S4. NMR spectrum of ABL in CD3OD (400 MHz for ^{1}H NMR, 100 MHz for ^{13}C NMR)

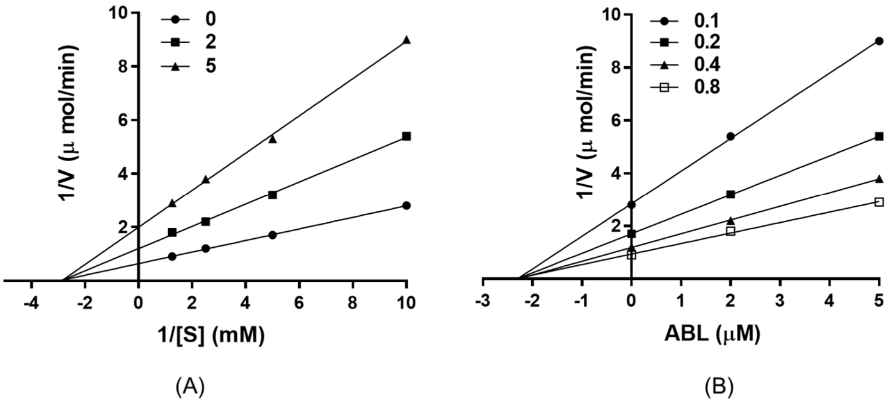

Figure S5. Kinetic study on HNE inhibition by ABL. (A) Double-reciprocal Lineweaver−Burk plots of ABL. The concentrations of ABL were 0, 2, and 5 μM; (B) Dixon plot of ABL. The concentrations of substrate were 0.1, 0.2, 0.4, and 0.8 mM.
